# Production of Flavours and Fragrances via Bioreduction of (4*R*)-(-)-Carvone and (1*R*)-(-)-Myrtenal by Non-Conventional Yeast Whole-Cells

**DOI:** 10.3390/molecules18055736

**Published:** 2013-05-16

**Authors:** Marta Goretti, Benedetta Turchetti, Maria Rita Cramarossa, Luca Forti, Pietro Buzzini

**Affiliations:** 1 Department of Agricultural, Environmental and Food Sciences & Industrial Yeasts Collection DBVPG, University of Perugia, Borgo XX Giugno 74, Perugia 06121, Italy; E-Mails: martago22@hotmail.it (M.G.); bturchetti@unipg.it (B.T.); 2 Department of Life Sciences, University of Modena & Reggio Emilia, via G. Campi 183, Modena 41125, Italy; E-Mail: mariarita.cramarossa@unimore.it

**Keywords:** biocatalysis, non-conventional yeasts (NCYs), ene-reductases, carbonyl reductases, monoterpenoids, (4*R*)-(−)-carvone, (1*R*)-(−)-myrtenal

## Abstract

As part of a program aiming at the selection of yeast strains which might be of interest as sources of natural flavours and fragrances, the bioreduction of (4*R*)-(−)-carvone and (1*R*)-(−)-myrtenal by whole-cells of non-conventional yeasts (NCYs) belonging to the genera *Candida*, *Cryptococcus*, *Debaryomyces*, *Hanseniaspora*, *Kazachstania*, *Kluyveromyces*, *Lindnera*, *Nakaseomyces*, *Vanderwaltozyma* and *Wickerhamomyces* was studied. Volatiles produced were sampled by means of headspace solid-phase microextraction (SPME) and the compounds were analysed and identified by gas chromatography–mass spectroscopy (GC-MS). Yields (expressed as % of biotransformation) varied in dependence of the strain. The reduction of both (4*R*)-(−)-carvone and (1*R*)-(−)-myrtenal were catalyzed by some ene-reductases (ERs) and/or carbonyl reductases (CRs), which determined the formation of (1*R,*4*R*)-dihydrocarvone and (1*R*)-myrtenol respectively, as main flavouring products. The potential of NCYs as novel whole-cell biocatalysts for selective biotransformation of electron-poor alkenes for producing flavours and fragrances of industrial interest is discussed.

## 1. Introduction

Biocatalysis represents an effective and sometimes preferable alternative to the standard synthesis of fine and/or optically active chemicals [1,2,3,4,5,6,7]. Overall, reactions catalysed by biological systems frequently exhibit high selectivity (chemo-, regio-, and stereo-selectivity) and can be considered environmentally acceptable because they typically occur under mild conditions. Both isolated enzymes and whole-cells can be used of as biocatalysts, but whole-cell biocatalysts are often preferable to the former because they are more convenient and stable sources of enzymes, with no need for costly enzyme purification and coenzyme addition. Moreover, because of the enzymes are kept within the natural environment of living cells, usually less enzyme inactivation occurs.

Flavours play a very important role in the quality perception of food and beverages, whereas fragrances represent an important part of soap and perfume industry [8,9,10]. Consumers have a strong preference for natural food additives over chemically synthesized ones. Both United States (US) [11] and European (EU) [12] laws have already labelled as “natural flavour” all those obtained from living cells, including Generally Regarded As Safe (GRAS) microorganisms [8]. Thus, products obtained by microorganisms and enzymes can be considered natural as long as natural raw materials are used. As a result, the “natural” label, allocated by EU and US food legislation, represents a strong marketing advantage [9,10].

Monoterpenes are one of the largest classes of flavouring compounds (over 400 different naturally occurring structures), and represent a valuable resource for the flavour and fragrance industry. The consumer requests for natural flavours and fragrances have encouraged a growing part of scientific community to study and develop novel biocatalysts for producing this class of molecules. Thus, the microbial and enzymatic biotransformation of some monoterpenoids, in particular a few ketones and aldehydes (e.g., carvone, menthol, citronellol, myrtenal and geraniol) into highly valuable flavouring derivatives is becoming of increasing interest because of their economic potential for the perfume, soap, food, and beverage industry [13,14,15,16,17,18,19,20,21,22]. Carvone is produced by over 70 different plants. It is found basically in two distinct stereoisomeric forms, which differ between them for their flavouring attributes: (i) (4*R*)-(−)-carvone, which is the principal constituent in spearmint (*Mentha spicata*) oil, and (ii) *S*-(+)-enantiomer, which is present in oils extracted from caraway (*Carum carvi*) seeds and from dill (*Anethum graveolens*) seeds. Biocatalytic transformation of carvone has recently been the focus of several studies, reporting that some enzymes may catalyze the reduction of C=C and C=O double bonds competitively, affording a mixture of saturated ketones, saturated alcohol and, more rarely, the allylic alcohol [14,20,23,24,25,26,27,28,29,30,31,32]. From an industrial point of view, carvone and related compounds are important flavours and fragrances of industrial interest [33]. In particular, due their high volatility, dihydrocarvones are potent inhibitors of bacteria and filamentous fungi, as well as prospective insect repellents [34], and have been used as chiral starting compounds in the synthesis of natural products (e.g., striatenic acid, pechueloic acid) [35,36,37], antimalarial drugs [38] and valuable chiral synthons [39,40]. Dihydrocarveols are valuable fragrance ingredients currently used in decorative cosmetics, fine fragrances, shampoos, soaps and other toiletries as well as in household products such as cleaners and detergents [41].

Although the use of yeast whole-cells as biocatalysts is a well-established practice, and a few yeast-catalysed processes have been even successfully scaled up from laboratory to the industrial level [5,42,43,44,45], if compared with other microbial domains (e.g., bacteria, filamentous fungi), the number of studies reporting the use of yeast whole-cells to catalyse the biotransformation of monoterpenes represents only a little percentage of the literature published so far [46]. To most people, yeasts are exemplified by the common baker’s yeast (taxonomically defined as belonging to the ascomycetous species *Saccharomyces cerevisiae*). This is in spite of the fact that this species represents only a small fragment of the huge taxonomic and metabolic diversity occurring in the yeast world. In fact, in recent decades, biotech-oriented research had paid its attention to the so-called non-conventional yeasts (NCYs), which demonstrated sometimes a superior biocatalytic aptitude than *S. cerevisiae* [43,44,47].

As a part of a program aiming at the selection of yeast strains as novel sources of natural flavouring molecules, the production of flavours and fragrances via bioreduction of (4*R*)-(*−*)-carvone and (1*R*)-(*−*)-myrtenal by whole-cells of non-conventional yeasts (NCYs), belonging to the genera *Candida*, *Cryptococcus*, *Debaryomyces*, *Hanseniaspora*, *Kazachstania*, *Kluyveromyces*, *Lindnera*, *Nakaseomyces*, *Vanderwaltozyma*, and *Wickerhamomyces* was studied.

## 2. Results and Discussion

**Scheme 1:**
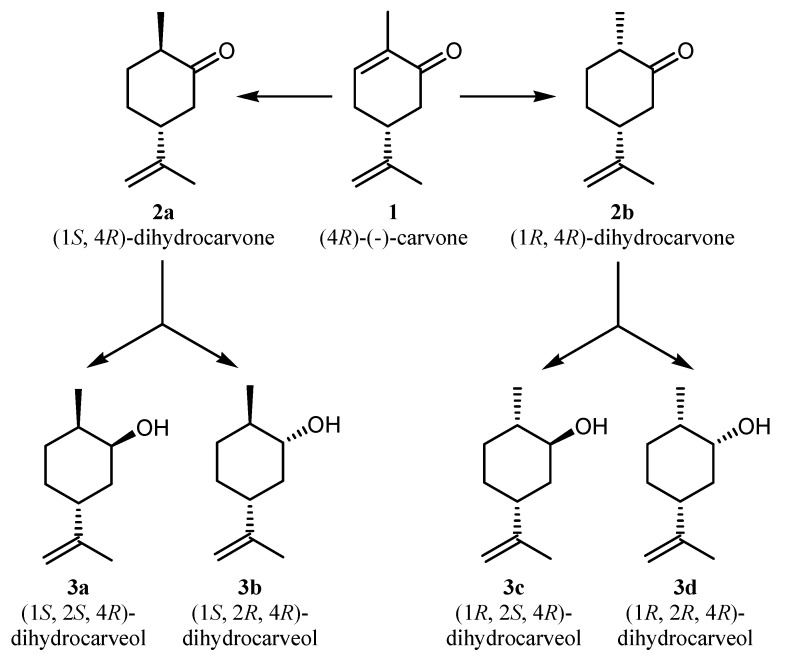
Bioconversion pathway of (1*R*)-(*-*)-myrtenal by whole-cells of NCYs

The biotransformations of the α,β-unsaturated ketone (4*R*)-(−)-carvone (**1**) catalyzed by whole-cells of NCYs in aqueous media were investigated. The possible reaction pathway is illustrated in Scheme 1. According to the proposed scheme, the biotransformation resulted in the reduction of the α,β-unsaturated C=C bond of the cyclic ketone, catalyzed by ene-reductases (ERs) associated to the yeast cells, to give two dihydrocarvones **2a**,**b**. The ER-catalysed reduction was thus followed by the subsequent reduction of the carbonyl group of both dihydrocarvone isomers, catalyzed by carbonyl reductases (CRs), which determined the formation of a mixture of four dihydrocarveols **3a**–**d** (Scheme 1).

Although the use of purified ERs needs an accompanying regeneration system for the nicotinamide cofactor [NAD(P)H] to close the catalytic cycle and improve the bioreduction efficiency, we assumed that lyophilized cells contain the needed recycling system except for the co-substrate. Accordingly, glucose was added because we found that this compound was the best co-substrate for co-factor recycling system [23]. In fact, from a quantitative point of view, the presence of glucose in the reaction mixture [acting as auxiliary substrate for NAD(P)H regeneration] resulted critical for enhance the % of conversion of precursor, in close agreement with current literature [23,48]. With no glucose addition, whole-cells of NCYs showed only a little ability to reduce *(*4*R)*-(−)-carvone. Only three strains gave acceptable results: *Hanseniaspora guilliermondii* DBVPG 6790 (conversion about 14%), *Lindnera amylophila* DBVPG 6346 (about 10%) and *Vanderwaltozyma polyspora* DBVPG 6243 (about 8%). The prevalent catalytic activity of whole-cells of NCYs was the ER-catalysed reduction of (4*R*)-(−)-carvone into a mixture of (1*R*,4*R*)- and (1*S*,4*R*)-dihydrocarvone, with a clear-cut preference towards the production of (1*R*,4*R*)-diastereomer. Only traces of dihydrocarveols **3a**-**d**, derived from the subsequent CR-catalysed reduction of the carbonyl group of (4*R*)-(−)-carvone were found. As expected, the addition of glucose to the reaction mixture, visibly increased the aptitude of some strains to reduce (4*R*)-(−)-carvone **1** (Table 1).

**Table 1:** Bioconversion of (4*R*)-(−)-carvone **1** into derivative products after 120 h by whole-cells of NCYs in the presence of glucose.

Species and strain	Conversion (mol %)	Products (mol %)					
		2b	2a	3b	3a	3d	3c
*C. maltosa* DBVPG 6021	12.93 ± 3.87	9.76 ± 4.49	0.55 ± 0.04	2.63 ± 0.93	0	0	0
*Cr. gastricus* DBVPG 6057	4.71 ± 0.15	3.73 ± 0.25	0.33 ± 0.03	0.66 ± 0.09	0	0	0
*C. oregonensis* DBVPG 6149	14.81 ± 4.46	6.69 ± 1.22	0.57 ± 0.08	7.40 ± 3.19	0	0.15 ± 0.13	0
*C. sake* DBVPG 6162	0.05 ± 0.05	0.05 ± 0.04	0.01 ± 0.01	0	0	0	0
*C. freyschussii* DBVPG 6208	1.09 ± 0.26	1.00 ± 0.23	0.01 ± 0.01	0.07 ± 0.02	0	0	0
*W. canadensis* DBVPG 6211	2.05 ± 0.24	0.36 ± 0.12	0	1.32 ± 0.15	0.13 ± 0.22	0.24 ± 0.22	0
*Cr. albidus* DBVPG 6237	0.74 ± 1.08	0.43 ± 0.56	0.30 ± 0.53	0	0	0	0
*Cr. terreus* DBVPG 6242	7.38 ± 12.43	7.24 ± 12.21	0.14 ± 0.22	0	0	0	0
*V. polyspora* DBVPG 6243	13.45 ± 17.77	13.35 ± 17.65	0.10 ± 0.13	0	0	0	0
*K. lodderae* DBVPG 6308	0.17 ± 0.04	0.17 ± 0.04	0	0	0	0	0
*L. amylophila* DBVPG 6346	7.79 ± 4.91	7.37 ± 4.64	0.42 ± 0.28	0	0	0	0
*K. exigua* DBVPG 6469	4.29 ± 0.83	2.87 ± 0.29	0.22 ± 0.04	1.20 ± 0.53	0	0	0
*Cr.s terreus* DBVPG 6685	0.94 ± 0.42	0.90 ± 0.40	0.05 ± 0.02	0	0	0	0
*Kaz. spencerorum* DBVPG 6746	11.38 ± 16.72	10.85 ± 16.23	0.46 ± 0.54	0.06 ± 0.10	0	0	0
*Kaz. spencerorum* DBVPG 6748	4.43 ± 0.28	3.98 ± 0.25	0.46 ± 0.03	0	0	0	0
*H. guilliermondii* DBVPG 6790	63.59 ± 15.44	62.11 ± 14.99	1.49 ± 0.45	0	0	0	0
*H. occidentalis* DBVPG 6798	6.26 ± 1.45	4.90 ± 1.05	0.41 ± 0.09	0.95 ± 0.33	0	0	0
*C. shehatae* DBVPG 6850	16.93 ± 3.20	7.59 ± 0.24	0.63 ± 0.02	8.71 ± 2.99	0	0	0
*K. marxianus* DBVPG 6854	0.24 ± 0.04	0.24 ± 0.04	0	0	0	0	0
*Kaz. africana* DBVPG 6934	1.44 ± 0.12	1.28 ± 0.11	0.16 ± 0.01	0	0	0	0
*N. bacillisporus* DBVPG 6945	0.33 ± 0.01	0.30 ± 0.02	0.03 ± 0.00	0	0	0	0
*N. bacillisporus* DBVPG 6962	1.27 ± 0.80	1.16 ± 0.70	0.10 ± 0.11	0	0	0	0
*D. nepalensis* DBVPG 7123	0.05 ± 0.01	0.05 ± 0.01	0	0	0	0	0
*D. coudertii* DBVPG 7124	0.020± 0.04	0.02 ± 0.04	0	0	0	0	0
*Kaz. naganishii* DBVPG 7133	12.25 ± 10.56	11.68 ± 10.26	0.57 ± 0.30	0	0	0	0

In particular, whole-cells of *H. guilliermondii* DBVPG 6790 exhibited a superior level of conversion (about 63%). Interestingly, the ERs associated with whole-cells of this strain exhibited a high chemoselectivity, thus resulting in the preferential reduction of the α,β-unsaturated C=C bond of (4*R*)-(−)-carvone to give almost exclusively (1*R*,4*R*)-dihydrocarvone **2b** (about 62%). No formation of dihydrocarveols was observed (Table 2). The high diasteroisomeric excess observed for this reaction (d.e. = 95%) suggested that the ERs of *H. guilliermondii* may attack hydrogen at the conjugated C=C double bond in a stereoselective way from the *si*-face at C-1 and re-face at C-6 by anti-addition.

**Table 2:** Bioconversion of (1*R*)-(−)-myrtenal **4** into derivative products after 120 h by whole-cells of NCYs

Strain	Conversion (mol %)	Main products (mol %)		
		5	6	7
*C. maltosa* DBVPG 6021	97.86 ± 0.47	95.56 ± 1.34	0	2.29 ± 0.98
*Cr. gastricus* DBVPG 6057	78.22 ± 20.3	65.64 ± 4.77	0	2.41 ± 0.15
*C. oregonensis* DBVPG 6149	98.82 ± 0.29	97.93 ± 0.33	0	0.89
*C. sake* DBVPG 6162	91.36 ± 1.24	0	74.80 ± 5.25	16.56 ± 4.81
*C. freyschussii* DBVPG 6208	100 ± 0.00	96.58 ± 1.87	0	4.15 ± 0.65
*W. canadensis* DBVPG 6211	52.58 ± 38.44	27.62 ± 47. 84	1.89 ± 1.68	23.07 ± 8.65
*Cr. albidus* DBVPG 6237	99.52 ± 0.82	2.26 ± 1.96	0	67.48 ± 38.82
*Cr. terreus* DBVPG 6242	38.49 ± 53.49	36.27 ± 55.37	0.58 ± 0.51	1.63 ± 1.49
*V. polyspora* DBVPG 6243	83.34 ± 4.43	79.06 ± 7.20	0	1.80 ± 0.78
*K. lodderae* DBVPG 6308	87.13 ± 1.79	84.20 ± 4.05	0	0.33 ± 0.19
*L. amylophila* DBVPG 6346	99.19 ± 0.17	0	0	82.65 ± 5.30
*K. exigua* DBVPG 6469	43.46 ± 37.32	17.65 ± 28.62	0.14 ± 0.24	25.67 ± 44.15
*Cr.s terreus* DBVPG 6685	0	0	0	0
*Kaz. spencerorum* DBVPG 6746	100 ± 0.00	98.68 ± 0.44	0	1.67 ± 0.39
*Kaz. spencerorum* DBVPG 6748	70.81 ± 0.92	70.23 ± 1.62	0	0.70 ± 0.81
*H. guilliermondii* DBVPG 6790	99.33 ± 1.16	64.20 ± 55.60	0	27.13 ± 28.72
*H. occidentalis* DBVPG 6798	78.53 ± 13.46	71.59 ± 12.25	0	6.10 ± 1.36
*C. shehatae* DBVPG 6850	58.50 ± 33.41	57.08 ± 32.76	0	1.46 ± 0.35
*K. marxianus* DBVPG 6854	32.65 ± 56.55	0	0	4.61 ± 5.32
*Kaz. africana* DBVPG 6934	40.87 ± 13.58	35.85 ± 53.03	0	5.96 ± 2.66
*N. bacillisporus* DBVPG 6945	51.64 ± 2.84	50.75 ± 2.58	0	1.15 ± 0.18
*N. bacillisporus* DBVPG 6962	97.02 ± 3.27	49.78 ± 57.48	0	11.16 ± 7.85
*D. nepalensis* DBVPG 7123	53.97 ± 51.15	53.97 ± 51.15	0	0
*D. coudertii* DBVPG 7124	54.41 ± 51.27	54.41 ± 51.27	0	0
*Kaz. naganishii* DBVPG 7133	0	0	0	0

Similarly to the biotransformation yields observed when *H. guilliermondii* was used, the addition of glucose to the reaction mixture increased the aptitude of some strains (otherwise practically incapable of catalysing the reaction) to reduce (4*R*)-(−)-carvone to levels of bioconversion > 10%: *Candida shehatae* DBVPG 6850 (17%), *Candida oregonensis* DBVPG 6149 (15%), *Candida maltosa* DBVPG 6021 and *V. polyspora* DBVPG 6243 (13%), *Kazachstania naganishii* DBVPG 7133 (12%) and *Kazachstania spencerorum* DBVPG 6746 (11%) (Table 1).

The increase of the bioconversion yield caused by glucose addition described above is not surprising. ERs are NAD(P)H-dependent flavoproteins and need an accompanying co-factor acting as regeneration system for the nicotinamide cofactor [NAD(P)H]. ERs often show a marked specificities for NADH or NADPH as co-factor, which allows to choose the best co-substrate for cofactor-recycling system on a case-to-case basis. A few authors [48] reported that yeast whole-cells contain only catalytic amounts of NAD(P)H, so its regeneration must take place by means of metabolism of an electron donor. In this framework, glucose could act as co-substrate for cofactor-recycling systems, thus exhibiting a significant efficacy in increasing the ER-catalysed reduction of (4*S*)-(−)-carvone. This result is consistent with current literature [44] that postulate that the presence of auxiliary substrates (e.g., glucose, formate, isopropanol,*etc.*) for cofactor-recycling system could reduce the problem of co-factor consumption and, therefore, greatly enhance the bioconversion yield.

Surprisingly, *C. shehatae* reduced the α,β-unsaturated C=C bond of (4*R*)-(−)-carvone in an chemoselective way divergent from that discussed above for *H. guilliermondii*, to give a slightly excess of (1*S*,4*R*)-dihydrocarvone (d.e. = 10%) (Table 2). This evidence could apparently support the hypothesis that the ERs associated with whole-cells of *C. shehatae* may attack hydrogen at the conjugated C=C double bond from both the *si*- and *re*-face at C-1. This could be due to the possibility of (4*R*)-(−)-carvone to flip into the enzymatic pocket of ER, or, alternatively, to the presence of a second ER (associated to whole-cells too) showing an opposite stereo-preference.

**Scheme 2:**
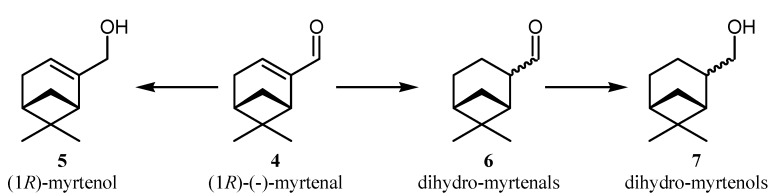
Bioconversion pathway of (1*R*)-(*-*)-myrtenal by whole-cells of NCYs

The biotransformations of the α,β-unsaturated aldehyde (1*R*)-(−)-myrtenal (**4**) catalyzed by whole-cells of NCYs were investigated. The possible reaction pathway is reported in Scheme 2. Based on the proposed scheme, ERs associated to whole-cells of NCYs catalysed the reduction of the α,β-unsaturated C=C bond of (1*R*)-(−)-myrtenal to give dihydromyrtenals **6**. The carbonyl group of the aldehyde was further reduced by CRs to give dihydromyrtenols **7**. A concurrent reaction, giving the production of (1*R*)-(*-*)-myrtenol **5** was also observed in some strains (Scheme 2).

Overall, NCYs used in this study showed good (in some cases excellent) aptitudes to biotransform (1*R*)-(−)-myrtenal (**4**) into derivative compounds, even in the absence of co-substrates for NAD(P)H regeneration (*i.e*., glucose): about one third of strains gave percentage of conversion ≥ 95%. Among them, *Candida freyschussii* DBVPG 6208 and *Kazachstania spencerorum* DBVPG 6746 converted 100% of the precursor (Table 3). Interestingly, in almost all cases, biocatalytic ability was prevalently related to the activity of CRs associated to whole-cells, affording myrtenol **5** as the main product of the bioconversions. On the contrary, the results obtained apparently suggest that (1*R*)-(−)-myrtenal (**4**) is not a good substrate for the ER-activity. In fact, only a few strains showed prevalent ER-catalysed asymmetric C=C bioreduction of the α,β-unsaturated aldehyde: they produced dihydromyrtenals **6**, which was then further reduced by CRs to give dihydromyrtenols **7**, that resulted the main products when *Cryptococcus albidus* DBVPG 6237 and *Lindnera amylophila* DBVPG 6346 were used as biocatalysts (Table 2).

Surprisingly, in some cases, (in particular when the biotransformation was promoted by *Cryptococcus gastricus* DBVPG 6057, *L. amylophila* DBVPG 6346, *Kluyveromyces marxianus* DBVPG 6854 and *Nakaseomyces bacillisporus* DBVPG 6962), the sum of (1*R*)-(−)-myrtenol **5** + dihydromyrtenals **6** + dihydromyrtenols **7** was apparently lower than % of conversion (Table 2). This could be due to the fact that GC-MS analysis did not reveal additional volatile by-products. This suggests that the actually lower biotransformation yield might be the result of the possible presence of side reactions. Among them, a special mention could be dedicated to myrtenoic acid, which was detected in little amounts (< 10%) in some strains and was revealed after extraction of samples with ethyl acetate. The production of this compound (not indicated in the bioconversion pathway reported in Scheme 2 may be due to a dismutation side reaction of aldehyde into the corresponding alcohol and carboxylic acid, promoted by an alcohol dehydrogenase (ADH), which was probably associated to whole-cells [49].

Among naturally occurring oxygen-containing monoterpene derivatives, (1*R*)-(−)-myrtenal is one of the most widespread flavouring compounds distributed in the *Asteraceae* (*Compositae*) family. It is a constituent of the cumin seed, juniper berry, pepper, peppermint, scotch spearmint and it is ubiquitous in the essential oils of flowers, stems, and leaves which possess a sweet and spicy cinnamon-like odour highly demanded as a key constituent of some fragrances [50]. Although only one study describing the biotransformation of myrtenal by algae has been so far published [51], the industrial importance of its structurally-related monoterpene alcohol derivative myrtenol is becoming greater and greater: this compound is a fragrance ingredient used in decorative cosmetics, fine fragrances, shampoos, toilet soaps and other toiletries as well as in non-cosmetic products such as household cleaners and detergents [52].

## 3. Experimental

### 3.1. Chemicals and Culture Media

*(*4*R)*-(−)-Carvone (5-isopropenyl-2-methyl-2-cyclohexenone) and (1*R*)-(−)-myrtenal [(5*R*)-6,6-dimethylbicyclo[3.1.1]hept-3-ene-4-carbaldehyde] were from Fluka (Sigma-Aldrich Co, St. Louis, MO, USA).

The following microbiological culture media were used: i) growth phase: YEPG: yeast extract 10 g L^−1^, peptone 10 g L^−1^, glucose 20 g L^−1^, agar 15 g L^−1^); ii) biotransformation phase: Yeast medium (YM): yeast extract 3 g L^−1^, malt extract 3 g L^−1^, peptone 5 g L^−1^, glucose 10 g L^−1^, pH 6.5. Ingredients of culture media were from Difco (BD, Franklin Lakes, NJ, USA).

### 3.2. Yeast Strains

Twenty-five NCY strains belonging to the genera *Candida*, *Cryptococcus*, *Debaryomyces*, *Hanseniaspora*, *Kazachstania*, *Kluyveromyces*, *Lindnera*, *Nakaseomyces*, *Vanderwaltozyma*, and *Wickerhamomyces*, were used (Table 3). They were preliminarily selected from amongst over 200 strains of environmental origin (representative of about 80 ascomycetous and basidiomycetous yeast species) for their ability to catalyse the biotransformation of *(*4*S)*-(*+*)-carvone (which was preliminarily used as model target substrate) into reduced products (dihydrocarvones and dihydrocarveols) [23]. All strains are conserved at the Industrial Yeasts Collection DBVPG of the University of Perugia, Italy [53] and are publicly available upon request. NCY strains were routinely maintained in frozen form (−80 °C). Working cultures were grown on YEPG agar slants at 25 °C.

**Table 3:** Salient information on NCY strains used in the present study.

Pecies and Strain	Origin	Location
*Candida maltosa* DBVPG 6021	Soil	Japan
*Cryptococcus gastricus* DBVPG 6057	Soil	New Zealand
*Candida oregonensis* DBVPG 6149	Frass of *Tsuga heterophylla*	USA
*Candida sake* DBVPG 6162	Soil	Sweden
*Candida freyschussii* DBVPG 6208	Wood pulp	Sweden
*Wickerhamomyces canadensis* DBVPG 6211	Wood pulp	Sweden
*Cryptococcus albidus* DBVPG 6237	Soil	Hungary
*Cryptococcus terreus* DBVPG 6242	Soil	Buthan
*Vanderwaltozyma polyspora* DBVPG 6243	Soil	South Africa
*Kazachstania lodderae* DBVPG 6308	Soil	South Africa
*Lindnera amylophila* DBVPG 6346	Frass of *Pinus taeda*	USA
*Kazachstania exigua* DBVPG 6469	Soil	South Africa
*Cryptococcus terreus* DBVPG 6685	Soil	ex USSR
*Kazachstania spencerorum* DBVPG 6746	Soil	South Africa
*Kazachstania spencerorum* DBVPG 6748	Gut of *Psychidae* larva	South Africa
*Hanseniaspora guilliermondii* DBVPG 6790	Trachea of bee	France
*Hanseniaspora occidentalis* DBVPG 6798	Soil	West Indies
*Candida shehatae* DBVPG 6850	Rain forest drosophilids	Brazil
*Kluyveromyces marxianus* DBVPG 6854	Rain forest drosophilids	Brazil
*Kazachstania africana* DBVPG 6934	Soil	Zimbabwe
*Nakaseomyces bacillisporus* DBVPG 6945	Exudate of *Quercus emoryi*	USA
*Nakaseomyces bacillisporus* DBVPG 6962	Exudate of *Quercus emoryi*	USA
*Debaryomyces nepalensis* DBVPG 7123	Soil	Nepal
*Debaryomyces coudertii* DBVPG 7124	Dropping of *Aptenodytes patgonica*	France
*Kazachstania naganishii* DBVPG 7133	Decaying leaves	Japan

### 3.3. Preparation of Lyophilized Whole-Cells Biocatalysts

Lyophilized NCY cells were obtained as previously described [23], with some modifications. Aliquots (200 μL) of 24 h cell suspensions, calibrated to A_580_ = 0.5 (average cell concentration = 10^6^ cells mL^−1^), were used to inoculate 5 mL of YM. Once the NCY biomass was reached following 48 h of incubation, cells and supernatants were separately harvested. NCY cells were washed three times by using 50 mM phosphate buffer (pH 6.5), centrifuged each time for 15 min at 4,000 rpm, snap frozen (−80 °C) and lyophilized for 48 h in a Lyophilizer Modulyo (Edwards, Sanborn, NY, USA).

### 3.4. Bioconversion Reactions

Thirty mg of lyophilized NCY cells were resuspended in 25 mL sterile vials containing 5 mL of 50 mM phosphate buffer (pH 6.5) + 50 mM glucose [acting as cofactor-recycling system for NAD(P)H]. Each substrate (final concentration 10 mM) was added and the vials were incubated on an orbital shaker (120 rpm) at 25 °C for 120 h. After incubation vials were sealed and frozen (−30 °C) until SPME + GC-MS analysis. All the reactions were carried out in triplicate. In order to determine whether substrates were spontaneously reduced in the absence of NCY cells, blank (cell-free) vials containing 50 mM phosphate buffer + 50 mM glucose and each substrate were analyzed at 120 h. As with previous samples, following incubation vials were sealed and frozen (−30 °C) until SPME + GC-MS analysis.

### 3.5. SPME and GC-MS Analyses

*(*4*R)*-(−)-Carvone and (1*R*)-(−)-myrtenal derivatives occurring in vial headspace after bioreduction reactions were detected by GC-MS using the solid-phase microextraction (SPME) sampling technique. Sealed vials containing the yeast suspensions were thawed by immersion in a silicon oil bath at 35 °C for 15 minutes. Headspace was analyzed using a 1-cm needle containing a fiber coated with 75 μM Carboxen/polydimethylsiloxane bonded to a flexible fused silica core (Supelco, Sigma-Aldrich Co, St. Louis, MO, USA). The needle was inserted into the vial through the septum and the fiber was exposed to headspace volatiles for 5 min at 30 °C. After direct desorption into the injector port at 280 °C for 5 min, the products were analyzed using a Hewlett Packard (Palo Alto, CA, USA) G1800C Series II gas chromatograph–mass spectrometer equipped with a HP-5 column (25 m × 0.2 mm, 0.5 μM film thickness) coated with (5%)-diphenyl-(95%)-dimethylpolysiloxane copolymer.

Compounds derived from biotransformation of *(*4*R)*-(−)-carvone and (1*R*)-(−)-myrtenal were identified by comparing their respective mass fragmentation patterns (EI, 70 eV) with the database library NIST05 (Varian MS Library Software, Palo Alto, CA, USA). A concentration of monoterpenes was measured in the vial headspace by an internal standard method in which thawing vial contents were spiked with 50 μL of a freshly prepared chlorobenzene solution (0.05 mg/mL in deionized water).

Myrtenoic acid obtained after (1*R*)-(−)-myrtenal bioconversion were detected via GC-MS after extraction with a solution of octanol (internal standard) in ethyl acetate 0.1% V/V (5 mL).

All the results were expressed as biotransformation yield, *i.e.*, % of substrate converted to a given derivative. The total ene-reductase (ER) or carbonyl reductase (CR) activities of NCY whole-cells were calculated as the sum of products obtained by reduction of a given substrate (catalyzed by ERs or CRs, respectively). All the results represented the average of three independent experiments, and the statistical significance of these average data was assessed via ANOVA.

When *(*4*R)*-(−)-carvone was used as substrate, the % of diasteroisomeric excess (d.e.) of *1R,4R-*isomers (or alternatively, *1S,4R-*isomers) was calculated as follow:

d.e. = [(*1R,4R-*isomers − *1S,4R-*isomers − or vice versa)/(*1R,4R-*isomers + *1S,4R-*isomers)] × 100

## 4. Conclusions

Although prokaryotic and eukaryotic microorganisms (including yeasts) are recognized to produce flavouring compounds potentially attractive for industry, the number of processes that can be considered truly competitive with chemical synthesis is at present limited, primarily because of their low yields [43]. Accordingly, as underlined by previous authors [46,53], the potential offered by yeast diversity, still far from being fully explored, could represent a noteworthy source of novel biocatalysts for the synthesis of flavours and fragrances. Moreover, the use of whole-cells is nowadays adequately accepted, not only as laboratory curiosity, but also in the industrial scale, because they are considered much more economical and easy-to-handle than purified enzymes [23,54,55].

In this paper, we selected some active NCYs for the stereoselective reduction of α,β-unsaturated C=C bond of cyclic ketones and aldehydes [namely (4*R*)-(−)-carvone and (1*R*)-(−)-myrtenal] to produce valuable flavouring derivatives. Overall, just one strain (*H. guilliermondii* DBVPG 6790) exhibited a good bioconversion yield of (4*R*)-(−)-carvone (about 63%), coupled with an excellent chemoselectivity yet (d.e. = 98%), compatible with its application as potential source of dihydrocarvone. Alternatively, some strain showed an excellent aptitude to totally transform (1*R*)-(−)-myrtenal to its flavoured derivative myrtenol (% of conversion ≥ 95%). Their further study for industrial scale-up is in progress.

## References

[1] Matsuda T., Yamanaka R., Nakamura K. (2009). Recent progress in biocatalysis for asymmetric oxidation and reduction. Tetrahedron Asymmetry.

[2] Bastos Borges K., de Souza Borges W., Durán-Patrón R., Tallarico Pupo M., Sueli Bonato P., González Collado I. (2009). Stereoselective biotransformations using fungi as biocatalysts. Tetrahedron Asymmetry.

[3] Hollmann F., Arends I.W.C.E., Holtmann D. (2011). Enzymatic reductions for the chemist. Green Chem..

[4] Hollmann F., Arends I.W.C.E., Buehler K., Schallmey A., Buehler K. (2011). Enzyme-mediated oxidations for the chemist. Green Chem..

[5] Winkler C.K., Tasnádi G., Clay D., Hall M., Faber K. (2012). Asymmetric bioreduction of activated alkenes to industrially relevant optically active compounds. J. Biotechnol..

[6] Patel R.N. (2011). Biocatalysis: Synthesis of key intermediates for development of pharmaceuticals. ACS Catal..

[7] Faber K. (2004). Biotransformations in Organic Chemistry.

[8] Lomascolo A., Stentelaire C., Aster M., Lesage-Meessen L. (1999). Basidiomycetes as new biotechnological tools to generate natural aromatic flavours for the food industry. TrendsBiotechnol..

[9] Krings U., Berger R.G. (1998). Biotechnological production of flavours and fragrances. Appl. Microbiol. Biotechnol..

[10] Serra S., Fuganti C., Brenna E. (2005). Biocatalytic preparation of natural flavours and fragrances. Trends Biotechnol..

[11] (1985). US Code of Federal Regulations.

[12] (1988). Council Directive 88/388/EEC on the approximation of the laws of the Member States relating to flavourings for use in foodstuffs and to source materials for their production. Official J. Eur. Union L.

[13] Lemos B.J., Dionsio A.P., Pastore G.M. (2009). Bio-oxidation of terpenes: An approach for the flavor industry. Chem. Rev..

[14] Cramarossa M.R., Nadini A., Bondi M., Messi P., Pagnoni U.M., Forti L. (2005). Biocatalytic reduction of (+)- and (–)-carvone by bacteria. C. R. Chim..

[15] Ponzoni C., Gasparetti C., Goretti M., Turchetti B., Pagnoni U.M., Cramarossa M.R., Forti L., Buzzini P. (2005). Biotransformation of acyclic monoterpenoids by *Debaryomyces sp.*, *Kluyveromyces. sp.*, and *Pichia. sp.* strains of environmental origin. Chem. Biodivers..

[16] King A.J., Dickinson J.R. (2000). Biotransformation of monoterpene alcohols by *Saccharomyces cerevisiae*, *Torulaspora*. *delbrueckii* and* Kluyveromyces. lactis.*. Yeast.

[17] Demyttenaere J.C.R., del Carmen Herrera M., de Kimpe N. (2000). Biotransformation of geraniol, nerol and citral by sporulated surface cultures of* Aspergillus*. *niger* and *Penicillium. sp.*. Phytochemistry..

[18] Demyttenaere J.C.R., de Pooter H.E. (1998). Biotransformation of citral and nerol by spores of *Penicillium. digitatum*. Flavour Fragrance J..

[19] Demyttenaere J.C.R., de Pooter H.E. (1996). Biotransformation of geraniol and nerol by spores of *Penicillium. italicum.*. Phytochemistry.

[20] Van Dyk M.S., van Rensburg E., Rensburg I.P.B., Moleleki N. (1998). Biotransformation of monoterpenoid ketones by yeasts and yeast-like fungi. J. Mol. Catal. B Enzymatic.

[21] Pinheiro L., Marsaioli A.J. (2007). Microbial monooxygenases applied to fragrance compounds. J. Mol. Catal. B Enzym..

[22] Brenna E., Fuganti C., Gatti F.G., Serra S. (2011). Biocatalytic methods for the synthesis of enantioenriched odor active compounds. Chem. Rev..

[23] Goretti M., Ponzoni C., Caselli E., Marchigiani E., Cramarossa M.R., Turchetti B., Buzzini P., Forti L. (2009). Biotransformation of electron-poor alkenes by yeasts: asymmetric reduction of (4S)-(+)-carvone by yeast enoate reductases. Enzyme Microb. Technol..

[24] Fryszkowska A., Toogood H., Sakuma M., Gardiner J.M., Stephens G.M., Scrutton N.S. (2009). Asymmetric reduction of activated alkenes by pentaerythritol tetranitrate reductase: specificity and control of stereochemical outcome by reaction optimisation. Adv. Synth. Catal..

[25] Goretti M., Branda E., Turchetti B., Cramarossa M.R., Onofri A., Forti L., Buzzini P. (2012). Response surface methodology as optimization strategy for asymmetric bioreduction of (4S)-(+)-carvone by *Cryptococcus gastricus*. Biores. Technol..

[26] Padhi S.K., Bougioukou D.J., Stewart J.D. (2009). Site-saturation mutagenesis of tryptophan 116 of *Saccharomyces pastorianus* Old Yellow Enzyme uncovers stereocomplementary variants. J. Am. Chem. Soc..

[27] Adalbjornsson B.V., Toogood H.S., Fryszkowska A., Pudney C.R., Jowitt T.A., Leys D., Scrutton N.S. (2010). Biocatalysis with thermostable enzymes: structure and properties of a thermophilic ‘ene’-reductase related to Old Yellow Enzyme. Chem. Bio. Chem..

[28] Hook I.L., Ryan S., Sheridan H. (2003). Biotransformation of aliphatic and aromatic ketones, including several monoterpenoid ketones and their derivatives by five species of marine microalgae. Phytochemistry..

[29] Hamada H., Yasumune H., Fuchikami Y., Hirata T., Sattler I., Williams H.J., Ian Scott A. (1997). Biotransformation of geraniol, nerol and (+)- and (−)-carvone by suspension cultured cells of *Catharanthus*. *roseus*. Phytochemistry..

[30] Shimoda K., Hirata T. (2000). Biotransformation of enones with biocatalysts—Two enone reductases from *Astasia. longa*. J. Mol. Catal. B Enzym..

[31] Dutra Silva V., Ugarte Stambuk B., da Graça Nascimento M. (2012). Asymmetric reduction of (4R)-(−)-carvone catalyzed by Baker’s yeast in aqueous mono- and biphasic systems. J. Mol. Catal. B Enzym..

[32] Chen X., Gao X., Wu Q., Zhu D. (2012). Synthesis of optically active dihydrocarveol via a stepwise or one-pot enzymatic reduction of (R)- and (S)-carvone. Tetrahedron Asymmetry.

[33] de Carvalho C.C.C.R., da Fonseca M.M.R. (2006). Carvone: why and how should one bother to produce this terpene. Food Chem..

[34] Porto C., Stuker C.Z., Mallmann A.S., Simionatto E., Flach A., do Canto-Dorow T., da Silva U.F., Dalcol I.I., Morel A.F. (2010). (*R*)-(−)-carvone and (1*R*,4*R*)-trans-(+)-dihydrocarvone from *Poiretia. latifolia* Vogel. J. Brazil. Chem. Soc..

[35] Aubin Y., Audran G., Monti H. (2006). Improved enantioselective synthesis of natural striatenic acid and its methyl ester. Tetrahedron Lett..

[36] Blay G., Garcia B., Molina E., Pedro J.R. (2007). Synthesis of (+)-pechueloic acid and (+)-aciphyllene. Revision of the structure of (+)-aciphyllene. Tetrahedron.

[37] Harrowven D.C., Pascoe D.D., Demurtas D., Bourne H.O. (2005). Total synthesis of (−)-colombiasin A and (−)-elisapterosin B. Angew. Chem. Int. Ed..

[38] Dong Y., McCullough K.J., Wittlin S., Chollet J., Vennerstrom J.L. (2010). The structure and antimalarial activity of dispiro-1,2,4,5-tetraoxanes derived from (+)-dihydrocarvone. Bioorg. Med. Chem. Lett..

[39] de Rouville H.P.J., Vives G., Tur E., Rapenne G., Crassous J. (2009). Synthesis and analytical resolution of chiral pyrazoles derived from (5*R*)-dihydrocarvone. New J. Chem..

[40] Krawczyk H., Sliwinski M., Kedzia J., Wojciechowski J., Wolf W.M. (2007). Asymmetric synthesis of enantiomerically pure 7-isopropenyl-4a-methyl-3-methyleneoctahydrochromen-2-ones. Tetrahedron Asymmetry.

[41] Bhatia S.P., Letizia C.S., Api A.M. (2008). Fragrance material review on dihydrocarveol (R,R,R). Food Chem. Toxicol..

[42] Demain A.L., Phaff H.J., Kurtzman C.P., Kurtzman C.P., Fell J.W. (1998). The Idustrial and Agricultural Significance of Yeasts. The Yeasts. A Taxonomic Study.

[43] Johnson E.A., Echavarri-Erasun C., Kurtzman C.P., Fell J.W., Boekhout T. (2011). Yeast biotechnology. The Yeasts. A Taxonomy Study.

[44] Buzzini P., Vaughan-Martini A., Rosa C.A., Gabor P. (2006). Yeast Biodiversity and Biotechnology. The Yeast Handbook. Biodiversity and Ecophysiology of Yeasts.

[45] Liese A., Seelbach K., Wandrey C. (2006). Industrial Biotransformations.

[46] De Carvalho C.C.R., da Fonseca M.M.R. (2006). Biotransformation of terpenes. Biotechnol. Adv..

[47] Wolf K., Breunig K., Barth G. (2003). Non Conventional Yeasts in Genetics, Biochemistry and Biotechnology.

[48] Van der Donk W.A., Zhao H. (2003). Recent developments in pyridine nucleotide regeneration. Curr. Opin. Biotechnol..

[49] Velonia K., Smonou I. (2000). Dismutation of aldehydes catalyzed by alcohol dehydrogenases. J. Chem. Soc. Perkin Trans. 1.

[50] Morgan G.K., Liu Z., Wene M.J., King D.F. (2007). Dryer-added fabric care articles.

[51] Noma Y., Takahashi H., Asakawa Y. (1991). Biotransformation of terpene aldehydes by *Euglena gracilis* Z. Phytochemistry..

[52] Bhatia S.P., McGinty D., Letizia C.S., Api A.M. (2008). Fragrance material review on myrtenol. Food Chem. Toxicol..

[53] The University of Perugia, Available online:. http://www.agr.unipg.it/dbvpg.

[54] Carballeira J.D., Alvarez E., Sinisterra J.V. (2004). Biotransformation of cyclohexanone using immobilized Geotrichum candidum NCYC49 - Factors affecting the selectivity of the process. J. Mol. Catal. B. Enzym..

[55] Goretti M., Ponzoni C., Caselli E., Marchegiani E., Cramarossa M.R., Turchetti B., Forti L., Buzzini P. (2011). Bioreduction of a,b-unsaturated ketones and aldehydes by non-conventional yeast (NCY) whole-cells. Biores. Technol..

